# What and How Much Do Children Lose in Academic Settings Owing to Parental Separation?

**DOI:** 10.3389/fpsyg.2017.01545

**Published:** 2017-09-11

**Authors:** Tania Corrás, Dolores Seijo, Francisca Fariña, Mercedes Novo, Ramón Arce, Ramón G. Cabanach

**Affiliations:** ^1^Forensic Psychology Institute, Universidade de Santiago de Compostela Santiago de Compostela, Spain; ^2^Political Science and Sociology, Universidade de Santiago de Compostela Santiago de Compostela, Spain; ^3^Faculty of Education Sciences and Sports, University of Vigo Vigo, Spain; ^4^Facultad de Fisioterapia, University of A Coruña A Coruña, Spain

**Keywords:** parental separation, school (mal)adjustment, aversion to learning, aversion to teachers, school (dis)satisfaction, indiscipline

## Abstract

The literature has firmly established an association between parental separation and school failure. Nevertheless, parental separation does not affect academic aptitudes. Thus, mediators explain such relationship. A field study was designed to identify and quantify damage in the mediating variables between parental separation and school failure (i.e., external school adjustment, aversion to institution, aversion to learning, aversion to instruction, aversion to teachers, indiscipline). A total of 196 children, classified into three age cohorts: 109 in level 1 (from 8 to 11 years), 46 in level 2 (from 12 to 14 years), and 41 in level 3 (15 or more years), were assessed in school adjustment and in underlying dimensions of school (mal)adjustment. The results showed significant effects of parental separation in school adjustment and in the underlying dimensions to maladjustment in the three classification levels. The magnitude of damage increased with age, i.e., small in level 1, moderate in 2, and large in 3. Damage in all the sub-dimensions underlying school (mal)adjustment was quantified. The implications of the results for the design and implementation of prevention and intervention programs for children from separated parents are discussed.

## Introduction

According to the Eurostat (2015) statistical data on separation and divorce in the EU-28, approximately 65% of adults live as couples (married or in consensual union) with approximately half ending in separation. Almost a million divorces and separations are recoded every year, around half of these involve children. Parental separation is linked to negative effects on children in terms of psychological adjustment, academic performance, behavioral disorders, self-concept, and social adjustment (Amato, 2001). The estimates on the average damage are around 17% in psychological adjustment; a 14.6% increase in the rate of academic failure (school repetition rate) and a 16.9% fall in academic performance; a rise in the mean rate of 13.2% in disruptive and 11.8% in aggressive behavior (behavioral disorders); a mean decrease of 32% in academic, 27% emotional, 22% physical, and 37% family self-concept; as well as in social adjustment as measured by a mean loss of 16% in self-control in social relations, and an increase of 21% in social withdrawal (Seijo et al., 2016). Moreover, children from broken homes have been found to convert psychological problems into physical symptoms, increasing the probability of developing gastrointestinal, genitourinary, dermatological, and neurological disorders due to parental breakup by 14.1, 7.7, 14.4, and 17.1%, respectively (Martinón et al., 2017). Both the clinical models (American Psychiatric Association, 2013), and the additive or accumulative deficit explanatory models of delinquency (Lösel et al., 1992) assert that damaged areas are interrelated and constitute a cluster of damages, making them highly resistant to intervention, and fostering persistent recidivism in maladjustment (Maruna, 2004; Hutchings et al., 2010). Moreover, some of these spheres may act as protective factors safeguarding from maladjustment (e.g., in academic performance, self-concept), whereas in others they reflect the level of damage such as psychological adjustment, behavioral disorders, and social adjustment. In particular, academic performance may prompt the risk or protect against violence and delinquency (Jolliffe et al., 2016), psychological distress (Lyndon et al., 2014), and dysfunctions in self-concept (Huang, 2011). The Reciprocal Effects Model provides a reasonable explanation for the relationship between self-concept and academic achievement sustaining that prior self-concept affects subsequent academic achievement, and conversely prior academic achievement impacts on subsequent academic self-concept, i.e., the influence is reciprocal (Marsh et al., 2005). This model has obtained substantial empirical evidence (Huang, 2011), and has been extended with success to the relation between other domains (Móller et al., 2011), fitting the interrelationship among the damaged areas resulting from parental separation.

Bearing in mind that parental separation does not affect the child’s aptitudes (e.g., IQ), mediators serve to explain the decrease in the damaged domains. The literature has identified beliefs and attitudes toward the educational system (Baker, 2006; Lee, 2016), school engagement (Wang and Holcombe, 2010), school environment (Norton, 2008; Roorda et al., 2011), and behavioral problems (Stipek and Miles, 2008) as the main mediators of academic achievement. Taking into account the literature and the fact that the probability of academic failure is directly associated to parental separation, a filed study was undertaken to assess the mediating variables of this effect and to quantify damage.

## Materials and Methods

### Participants

A total of 196 children from separated parents participated in the study. Participants were classified by the instrument measure (TAMAI) according to the following age cohorts: 109 participants in level 1, 56.9% females and 43.1% males, aged 8–11 years (*M* = 9.94, *SD* = 1.04); 46 participants in level 2, 54.3% males and 45.6% females, aged 12–14 years (*M* = 13.20, *SD* = 0.78); and 41 participants in level 3, 51.2% females and 48.8% males, aged 15 years or more (*M* = 16.10, *SD* = 1.05).

### Procedure

Participants were recruited from the pediatric catchment area of Santiago de Compostela, a city in North-western of Spain. Pediatricians were contacted to access the children from separated parents. To measure the chronic effects of separation, a minimum 1-year of parental separation was established. Most of the children (>90%) identified as coming from separated parents participated voluntarily in the study. Informed consent was obtained from parents, and children participated voluntarily. Data were processed in compliance with the Spanish Data Protection Law to guarantee the privacy and anonymity of participants and their families.

*Post hoc* analysis of design sensitivity (1-β) for a mean comparison with a test value, a moderate effect size (*d* = 0.5), and a Cronbach’s alpha of 0.05 showed a design sensitivity (i.e., the probability of finding significant differences if they exist) for a sample size of 109 subjects (level 1), 46 subjects (level 2), and 41 subjects (level 3) of 99.9, 95.5, and 93.3%, respectively.

### Measurement Instrument

Maladjustment in the school setting was measured by the TAMAI (Mutifactorial Self-Administered Test of Child Adjustment) by Hernández-Guanir (2015). The instrument divided the children into three levels according to differences in the underlying maladjustment dimensions mediated by the school level and age of the children: level 1 – from 8 to 11 years, studying 3rd, 4th, or 5th year of primary education in the Spanish school system; level 2 – from 12 to 14 years, studying 6th year of primary education, and 1st and 2nd year of secondary education; and level 3 – 15 years or more, studying 3rd or 4th year of secondary education. The underlying dimensions for school maladjustment at level 1 are: external school maladjustment (i.e., low commitment and indiscipline); aversion to the institution (i.e., toward teachers and school); and aversion to learning (i.e., toward studying and knowledge). For level 2, the sub-dimensions are aversion to instruction consisting of hypo-commitment (i.e., low commitment to learning), hypo-motivation (i.e., little interest in learning), and aversion to teachers (i.e., dissatisfaction with teachers); and indiscipline (i.e., disruptive classroom behavior). For level 3, the sub-dimensions are aversion to instruction consisting of hypo-commitment (i.e., low commitment to learning), hypo-motivation (i.e., little interest in learning), school dissatisfaction (i.e., dissatisfaction in classroom and college), and aversion to teachers (i.e., dissatisfaction with teachers); and indiscipline (i.e., disruptive classroom behavior). The internal consistency obtained for the participants in the study was: Cronbach’s alpha of 0.86 for the whole sample; 0.71 for level 1 (sub-dimensions: external school adjustment = 0.79; aversion to instruction = 0.71; and aversion to learning = 0.69); 0.79 for level 2 (sub-dimensions: hypo-commitment = 73; hypo-motivation = 0.81; aversion to teachers = 0.80; indiscipline = 0.72); and 0.83 for level 3 (sub-dimensions: aversion to instruction = 0.89; hypo-commitment = 0.75; hypo-motivation = 0.70; school dissatisfaction = 0.71; aversion to teachers = 0.68; indiscipline = 0.84).

### Data Analysis

The mean for the sample of children from separated parents was compared with the mean adjustment of the normative population (test value) provided in the instrument manual. As for the effect sizes Cohen’s *d* was computed, being the confidence intervals for *d* derived from with Hunter and Schmidt’s (2015) formula to estimate the generalization of the results to other samples. Additionally, the BESD statistic (Rosnow and Rosenthal, 1996) was calculated to quantify mean injury and the intervals of injury for 95% of subjects. In order to contrast differences in damage among levels, the differences among the correlations were computed (Cohen, 1988).

## Results

### General Damage in School Adjustment

The results (see **Table 1**) show significant positive effects (i.e., separation was related to high maladjustment) in maladjustment at school in the three child classification levels, with a small effect size in level 1, moderate in 2, and large in 3. Notwithstanding, these results are not generalizable (when 95% CIs for *d* include zero, the results may not be generalized) to the entire population of children from separated parents. As for the magnitude of injury, mean injury was 21, 29, and 38%, ranging from 2.3 to 38.3% at level 1; 38.8 to 76.5% at level 2; and 8.2 to 61.6% at level 3. Comparatively, the lower limit of damage was significantly higher at level 2, 38.8% (*r* = 0.388) in contrast to level 1, 2.3% (*r* = 0.023), *q*_s_ = 0.392, *p* < 0.05. Whereas the upper limit of damages was significantly lower at level 1, 38.3% (*r* = 0.383), as compared to level 2, 76.5% (*r* = 0.765), *q*_s_ = 0.596, *p* < 0.01.

**Table 1:** One sample *t*-test for scholar maladjustment by level of studies.

Variable	*t* (*df*)	*M*_sf_	*tv*	*d* (95% CI*_d_*)	*r* (95% CI*_r_*)
Level 1	4.35 (108)^∗∗∗^	7.07	4.0	0.42 (-3.581, 4.421)	0.21 (0.023, 0.383)
Level 2	4.15 (45)^∗∗∗^	10.59	6.0	0.61 (-3.492, 4.712)	0.29 (0.388, 0.765)
Level 3	5.19 (40)^∗∗∗^	13.46	7.5	0.81 (-3.373, 4.994)	0.38 (0.082, 0.616)

*^∗∗∗^p < 0.001; M_sf_: mean of the separated family group; tv: test value (mean of the normative population)*.

### Analysis of the Sub-dimensions of the School Maladjustment

As for level 1 (i.e., children from 8 to 11 years), the results (see **Table 2**) showed significantly higher external school maladjustment, aversion to the institution, and aversion to learning. Nevertheless, these results may not be generalized to the whole population of children from separated parents (CIs for *d* includes 0). That is, parental separation may have adjustment effects for some samples. These may be up to more than three standard deviations (see the CIs lower limits which are related to adjustment). The average amount of damages was 21, 13, and 25% for external school maladjustment, aversion to the institution, and aversion to learning, respectively. While damages were equal in all the sub-dimensions (CIs for *r* overlap), for external school maladjustment and aversion to learning were positive and significant (CIs of *r* do not include 0), and not significant for aversion to the institution (negative CI lower limit), meaning that for some children separation diminished the aversion to the institution (positive effects).

**Table 2:** One sample *t*-test for the sub-dimensions of scholar maladjustment at level 1.

Variable	*t* (*df*)	*M*_sf_	*tv*	*d* (95% CI*_d_*)	*r* (95% CI*_r_*)
External school maladjustment	4.49 (108)^∗∗∗^	2.27	1.0	0.43 (-3.306, 4.923)	0.21 (0.023, 0.383)
Aversion to the institution	2.82 (108)^∗∗^	1.47	1.0	0.26 (-3.713, 4.233)	0.13 (-0.060, 0.310)
Aversion to learning	5.47 (108)^∗∗∗^	3.34	1.5	0.52 (-3.503, 4.543)	0.25 (0.065, 0.418)

*^∗∗^p < 0.01; ^∗∗∗^p < 0.001; M_sf_: mean of the separated family group; tv: test value (mean of the normative population).*

At level 2 (from 12 to 14 years) (see **Table 3**), significant and positive effects, that is, higher levels of maladjustment, were observed in aversion to instruction, hypo-commitment, hypo-motivation, and aversion to teachers. No effects were registered in indiscipline. However, the results may not be generalized to the entire population of children (CIs for *d* include 0). The average damage registered in aversion to instruction, hypo-commitment, hypo-motivation, and aversion to teachers was 33, 29, 29, and 28%, respectively. Interestingly, the lower limits for hypo-motivation and hypo-commitment were 0 and negative for aversion to teachers, meaning that for some children there were no effects or adjustment effects (negative scores indicate adjustment, and positive scores maladjustment).

**Table 3:** One sample *t*-test for the sub-dimensions of scholar maladjustment at level 2.

Variable	*t* (*df*)	*M*_sf_	*tv*	*d* (95% CI*_d_*)	*r* (95% CI*_r_*)
Aversion to instruction	4.66 (45)^∗∗∗^	9.11	5.0	0.69 (-3.438, 4.818)	0.33 (0.044, 0.566)
Hypo-application	4.10 (45)^∗∗∗^	2.83	1.5	0.61 (-3.492, 4.712)	0.29 (0.000, 0.535)
Hypo-motivation	4.06 (45)^∗∗∗^	4.70	3.0	0.60 (-3.499, 4.699)	0.29 (0.000, 0.535)
Aversion to teachers	3.90 (45)^∗∗∗^	1.59	0.5	0.58 (-3.514, 4.674)	0.28 (-0.011, 0.527)
Indiscipline	1.62 (45)	1.48	1.0	0.24 (-3.785, 4.265)	0.12 (-0.176, 0.396)

*^∗∗∗^p < 0.001; M_sf_: mean of the separated family group; tv: test value (mean of the normative population).*

As for level 3 (≥15 years), the results (see **Table 4**) revealed that children from separated parents exhibited significantly higher maladjustment manifested by aversion to instruction, hypo-commitment, hypo-motivation, school dissatisfaction, aversion to teachers, and indiscipline. Once again, results may not be generalized to children from the separated parent population. In relation to damage quantification, the observed average was of 38, 30, 42, 33, 23, and 21% for aversion to instruction, hypo-commitment, hypo-motivation, school dissatisfaction, aversion to teachers, and indiscipline, respectively. Notwithstanding, the damage for hypo-commitment, aversion to teachers, and indiscipline was not significant as the CIs lower limits were negative, meaning that for some children more adjustment effects on these variables were registered.

**Table 4:** One sample *t*-test for the sub-dimensions of scholar maladjustment at level 3.

Variable	*t* (*df*)	*M*_sf_	*tv*	*d* (95% CI*_d_*)	*r* (95% CI*_r_*)
Aversion to instruction	5.26 (40)^∗∗∗^	12.34	7.0	0.82 (-3.367, 5.001)	0.38 (0.082, 0.616)
Hypo-application	4.08 (40)^∗∗∗^	4.87	3.0	0.63 (-3.490, 4.750)	0.30 (-0.060, 0.310)
Hypo-motivation	5.93 (40)^∗∗∗^	5.02	2.5	0.92 (-3.309, 5.149)	0.42 (0.129, 0.644)
School dissatisfaction	4.58 (40)^∗∗∗^	0.63	0.0	0.71 (-3.437, 4.857)	0.33 (0.025, 0.579)
Aversion to teachers	3.05 (40)^∗∗^	1.80	1.0	0.47 (-3.607, 4.547)	0.23 (-0.074, 0.502)
Indiscipline	2.85 (40)^∗∗^	1.41	0.5	0.44 (-3.630, 4.510)	0.21 (-0.104, 0.486)

*^∗∗^p < 0.01; ^∗∗∗^p < 0.001; M_sf_: mean of the separated family group; tv: test value (mean of the normative population).*

## Discussion

Although the data processing design took into account the generalization of the results, this study entails three limitations derived from the design of the field study. First, the study design was transversal (versus longitudinal), thus the evolution of damages throughout the child’s development have not been ascertained. Second, the mean effects in children have been considered without taking into account the moderators of this relationship such as the degree of pre-separation and post-separation conflict, the child’s gender, and co-parenting. Third, the responses of the children were prone toward biased over-reporting (Arce et al., 2015b) and defensiveness (Arce et al., 2015a) given that the children were immersed in a process involving parental disputes, e.g., judicial litigation, parental interference, and conflict of loyalties.

Bearing in mind the limitations of this study, the following conclusions for mediating variables between parental separation and academic achievement, for quantifying damages may be drawn from the results. First, in general parental separation had negative effects on the children’s school adjustment. The magnitude of these negative effects increased with age, being small in level 1, moderate in 2, and large in 3. This tendency was equivalent, compatible, and complementary to the hypothesis of an escalating natural trajectory toward antisocial behavior (e.g., disruptive, violent, delinquent). In other words, the effects on maladjustment follow the natural tendency of increasing with the child’s development, i.e., the older the child the greater the negative effects (Hawley, 2003; Arce et al., 2011). The interrelationship between school (mal)adjustment and antisocial behaviors is such that school adjustment (e.g., high academic achievement, positive attitude to school) serves as a robust protective factor against violence (Jolliffe et al., 2016), whereas school maladjustment is one of the central eight antisocial risk factors (Andrews and Bonta, 2010). Moreover, school maladjustment is closely linked to a general and persistent life-long maladjustment trajectory (Fontaine et al., 2009; Arce et al., 2010; American Psychiatric Association, 2013). Second, the results are not generalizable to the global population of children from broken homes. The lack of generalization implies there were moderators of this relation, i.e., the existence of variables mediating the results of the effects. The most important moderator may be conflict, both in pre- and post-separation (Arce et al., 2005; Turner and Kopiec, 2006; Lacey et al., 2014). Moreover, other relevant moderators may be paternal school involvement, parent–child relationship, financial (in)stability, and decision-making concerning legal custody (Pruett et al., 2003; Bernard et al., 2015; Berryhill, 2017). Third, with the exception of the indiscipline sub-dimension in level 2, damage was significant in all of the sub-dimensions and levels. In other words, damage comprises a set of variables underlying academic performance, i.e., in attitudes (i.e., negative attitudes toward school and learning), the school environment (i.e., school dissatisfaction, dissatisfaction with teachers), engagement (low motivation and commitment), and behavioral problems (disruptive behavior, indiscipline). Thus, academic failure is an underlying outcome of these damages, and to cope with academic failure interventions should be targeted to repair them. Fourth, the mean magnitude of injury in school adjustment ranged from small (0.10 > *r* < 0.30) to moderate (0.30 > *r* < 0.50), and for particular children it fluctuated from negative effects in maladjustment (i.e., more adjustment) to no or large effects (*r* > 0.50) in maladjustment. The results are in line with the previous literature asserting that parental separation has no effect on many children, whereas for others it derives in positive or negative outcomes (Amato and Anthony, 2014), with a mean negative effect for the total population of children from broken homes (Amato, 2001). Fifth, the underlying sub-dimensions to school maladjustment fluctuated among levels. Thus, according to the need principle of the Risk-Need-Responsive model (Andrews and Bonta, 2010), which meta-analyses have found to be valid for intervention (Hanson et al., 2009; Koehler et al., 2013), interventions should target these sub-dimensions.

In terms of the damage detected and its magnitude, the results of this study underscore the need for implementing damage prevention and intervention programs for children from separated parents. Thus, future research should be directed to profile and assess the moderators of adjustment and maladjustment effects to derive protective and risk factors for evidence-based prevention and intervention programs.

## Ethics Statement

This study was approved by the Clinical Research Ethics Committee of the Autonomous Community of Galicia (Spain). Data were processed in compliance with the Spanish Data Protection Law.

## Author Contributions

The authors TC, DS, FF, MN, RA, and RGC have made a substantial, direct and intellectual contribution to the work.

## Conflict of Interest Statement

The authors declare that the research was conducted in the absence of any commercial or financial relationships that could be construed as a potential conflict of interest.
